# Characterisation of the Behavioural Effects of a Thoracic Squeeze in Healthy Newborn Piglets

**DOI:** 10.3390/ani11082465

**Published:** 2021-08-22

**Authors:** Sophia E. Holdsworth, Nikki J. Kells, Kirsty L. Chidgey, Emilie Vallée, Neil Ward, David J. Mellor, Ngaio J. Beausoleil

**Affiliations:** 1 Animal Welfare Science and Bioethics Centre, School of Veterinary Science, Massey University, Private Bag 11-222, Palmerston North 4442, New Zealand; S.Holdsworth@massey.ac.nz (S.E.H.); N.J.Kells@massey.ac.nz (N.J.K.); N.Ward@massey.ac.nz (N.W.); D.J.Mellor@massey.ac.nz (D.J.M.); 2 School of Agriculture and Environment, Massey University, Private Bag 11-222, Palmerston North 4442, New Zealand; K.L.Chidgey@massey.ac.nz; 3 EpiCentre, Massey University, Private Bag 11-222, Palmerston North 4442, New Zealand; E.Vallee@massey.ac.nz

**Keywords:** thoracic squeeze, low vigour, chest squeeze, reflex responses, loss of posture, cessation of movement, neonatal piglets, tonic immobility

## Abstract

**Simple Summary:**

Firmly squeezing the chests of newborn foals and calves that are showing abnormal behaviours after birth causes them to enter a less-responsive state, characterised by lying down with eyes closed and no limb movements. Once the squeeze is removed, the newborns immediately ‘wake up’ and begin to display more normal behaviours. This response to the thoracic squeeze has also been observed in healthy, normally behaving foals. However, no studies have looked at the effects of the thoracic squeeze in healthy newborns of other mammalian species. We aimed to characterise the behavioural responses of healthy newborn piglets to a thoracic squeeze using the following two methods: a soft fabric rope, or a purpose-made inflation cuff. Behavioural data indicated that all piglets initially became less responsive, with reduced or absent reflex responses to a toe pinch or touch of the eyelid observed in over half of the piglets. The piglets squeezed with the inflation cuff appeared to enter a less-responsive state faster than the piglets squeezed with the rope. These findings suggest that the piglets responded to the thoracic squeeze in a similar way to healthy foals and that this may be a response conserved across multiple precocial mammalian species. Furthermore, the squeeze was found to be safe for inducing a less-responsive state in healthy piglets. This study provides a foundation for exploring the mechanisms underlying the responses to the thoracic squeeze and potential applications whilst performing husbandry procedures.

**Abstract:**

A thoracic squeeze has been observed to cause both healthy and low vigour neonatal foals to enter a ‘less-responsive state’, characterised by loss of posture, eye closure and cessation of movement, from which they rapidly recover to express normal healthy behaviours when the squeeze is released. To date, there have been no systematic studies characterising the responses of healthy neonates of other mammalian species to a thoracic squeeze. We describe the responses of healthy newborn piglets (*n* = 17) to a standardised application of the thoracic squeeze and evaluate the effect of the method of squeeze application on the response. Neonatal piglets were squeezed around the chest with either a soft fabric rope as has been used in foals (*n* = 8) or a novel purpose-made inflation cuff (*n* = 9). Both methods were effective at inducing a less-responsive behavioural state in all piglets, with neural reflexes reduced or absent in over half of them. The inflation cuff appeared to induce the less-responsive state faster than the rope, and more piglets squeezed with the cuff remained in this state for the full 10-min squeeze. These findings suggest that the behavioural response of foals to thoracic squeezing can be generalised to neonates of other precocial mammalian species. This initial study provides a foundation for further research using the inflation cuff to explore mechanisms underlying the thoracic squeeze and ways in which it may be applied whilst performing husbandry procedures.

## 1. Introduction

Neonatal precocial mammals must express a specific pattern of behaviours soon after birth to ensure their survival [1,2,3]. These behaviours progress from uncoordinated to coordinated locomotion, searching for a functional teat and sucking colostrum [4]. Any underlying issue that impairs the expression of this pattern of behaviours increases the risk of morbidity and mortality [5]. Such impairment is recognised as ‘low vigour’, which is characterised by a delay in the onset of, or an inability to express, survival-related behaviours [4]. These abnormal behaviours include an inability to stand or walk, no affinity for the dam and no sucking reflex [6,7]. Early life mortality for piglets ranges from 10–20%, with low vigour thought to make up nearly 30% of piglet mortalities in the first week after birth [4,8]. Often, low vigour is related to pathophysiology (such as acute or chronic hypoxia), genetic disorders, physical malformations or underlying disease [4]. However, some neonatal farm mammals that are apparently healthy at birth also appear to have low vigour and, without intervention, often die. 

A therapeutic approach that is gaining popularity for these otherwise healthy low vigour animals is the ‘thoracic squeeze’ [6,9]. This involves applying a squeeze to the thorax of the neonate by looping a rope three times around the chest and firmly tightening. The squeeze reportedly causes these neonates to enter a ‘less-responsive state’, from which they rapidly recover to express normal healthy behaviours when the squeeze is released [7,10]. These healthy behaviours include unassisted standing and walking, successful teat seeking and unassisted drinking of colostrum and milk [6,10]. The mechanism by which the squeeze affects these low vigour neonates is not known.

The thoracic squeeze has also been shown to elicit a less-responsive state in healthy newborn foals [9]. Preliminary research found a distinctive change in behaviour, with foals entering an apparently somnolent (sleep-like) state accompanied by a decrease in muscle tone [9]. Small decreases in heart rate and respiratory activity also occurred, with a switch to abdominal breathing soon after the application of the rope. The removal of the squeeze resulted in a rapid return to normal healthy behaviours and normal cardiorespiratory function.

To date, there have been no systematic studies of the effects of the thoracic squeeze on healthy or low vigour piglets. Before applying the thoracic squeeze as a therapy for low vigour in piglets, there is a need to first demonstrate that a standardised application of the squeeze has no adverse effects on healthy neonates of this species. A detailed characterisation of responses is needed to inform an understanding of the mechanisms of the thoracic squeeze in both healthy and low vigour animals. Moreover, this technique may have practical applications for animal production industries. 

In addition, it is difficult to apply a uniform squeeze using three loops of a rope, and the features of rope application required to elicit a less-responsive state are difficult to measure and standardise, particularly in small neonates such as piglets. Thus, there is value in exploring whether other methods of squeeze application may provide a more uniform squeeze with a more standardised application. 

The aim of this study was to describe the responses of healthy neonatal piglets to a standardised application of the thoracic squeeze. A secondary aim was to compare piglets’ responses to squeeze application using a conventional rope and a novel purpose-made inflation cuff.

## 2. Materials and Methods

The study was undertaken on a commercial piggery in the Manawatū region, New Zealand. All procedures were approved by the Massey University Animal Ethics Committee (MUAEC Protocol 19/06). 

### 2.1. Selection of Animals

Seventeen healthy Large White X Landrace piglets were selected between 12 and 36 h old, using the inclusion criteria described below. Piglets were selected from litters of 12 or fewer to reduce the likelihood of applying the squeeze to individuals with underlying pathophysiological impairment, such as placental insufficiency or intrapartum hypoxaemia, which are more common in larger litters [11,12,13]. Within the litter, piglets that appeared to be the most vigorous (e.g., showing behaviours such as walking, teat seeking and sucking milk) were chosen. Piglets that were small, apparently weak or had abnormal conformation (e.g., protruding forehead, shorter snout, leg deformities) were rejected to avoid any confounding impacts of underlying health problems [12].

The inclusion criteria for selection were normal locomotion, social behaviour, body posture and rectal temperature (as defined in Table 1). Piglets had to stand, walk, vocalise or move their head or limbs when undisturbed or approached by researchers. Normal social behaviour was demonstrated by seeking contact with the dam or littermates, or reciprocating social interactions made by the dam or littermates. Normal body posture required a curled tail and relaxed, forward-facing ears (K. Chidgey, personal communication, April 2019). Rectal temperature had to be within the normal range of 38–39 °C [14]. Selected piglets were identified with coloured spray marker applied at either the base of the neck or at the tail root. 

### 2.2. Procedures

Due to the limited availability of litters within the desired age range, up to 6 piglets from a single litter were selected. The first four piglets were taken from a single litter and were squeezed with the rope due to initial difficulties with the inflation cuff device. Thereafter, piglets were alternately assigned to either the rope or the cuff treatment. Overall, eight piglets were squeezed using the rope method and nine using the cuff method over a total of four litters. Each piglet was squeezed only once to prevent a cumulative response. All procedures were undertaken within the farrowing room, on the side of the sow’s pen whilst maintaining standard conditions with regard to lighting, noise and temperature as specified in the Animal Welfare (Pigs) Code of Welfare [15].

For application of the squeeze, each piglet was lifted from the floor of the farrowing pen and placed onto a wooden board lined with foam beside the pen. The rope or cuff was firmly secured around the thorax and the squeeze applied as described below. A timer was started upon application of the squeeze and the piglet’s eyes were covered with a dark cloth once they closed to reduce light stimulation. The squeeze was applied until the piglet met the discontinuation criteria outlined below or the maximum time of 10 min was reached. This duration was based on previous reports of the 20 min squeeze applied to foals, which is apparently related to the time spent in the birth canal during birth [6,9]. Time spent in the birth canal for piglets is between 12 and 20 min [11,16,17]. Thus, choosing a duration that was less than that of birth ensured that the risks associated with a protracted squeeze were mitigated. After the squeeze was completed or discontinued, each piglet was immediately replaced in the farrowing pen with the sow and monitored for at least 5 min to ensure it had recovered from the squeeze and displayed the behaviours described in the inclusion criteria.

### 2.3. Treatments

**Figure 1:**
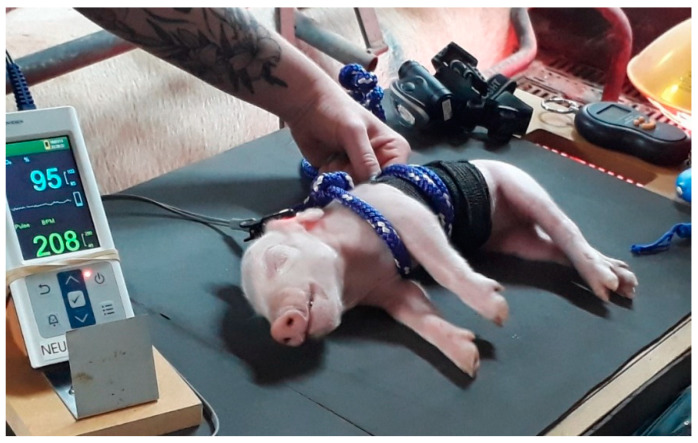
Rope squeeze applied to the thorax of a piglet, with vet wrap secured underneath the rope and pulse oximetry electrode attached to the ear.

The rope squeeze was applied using a soft polypropylene fabric rope of 1 cm diameter and approximately 1.5 m in length. The piglet was held in a standing position while the squeeze was applied. Vet wrap was first placed around the thorax to prevent pinching or tearing of the skin. A bowline knot was secured at one end and the rope was placed between the piglet’s front legs and looped through the knot at the wither (Figure 1). The rope was looped twice more around the thorax in the same direction and secured with half hitches, ensuring that each loop was evenly spaced across the thorax and would not slip onto the abdomen. Once the loops were in place, each loop was manipulated to squeeze firmly against the thorax. Each loop was then tightened until a finger could not be fitted between the vet wrap and the rope. The free end of the rope was pulled taut and held at the half hitch of the last loop. The timer was started when the most cranial loop of the rope had been tightened. Release of the squeeze was achieved by loosening and removing the rope from the thorax.

**Figure 2:**
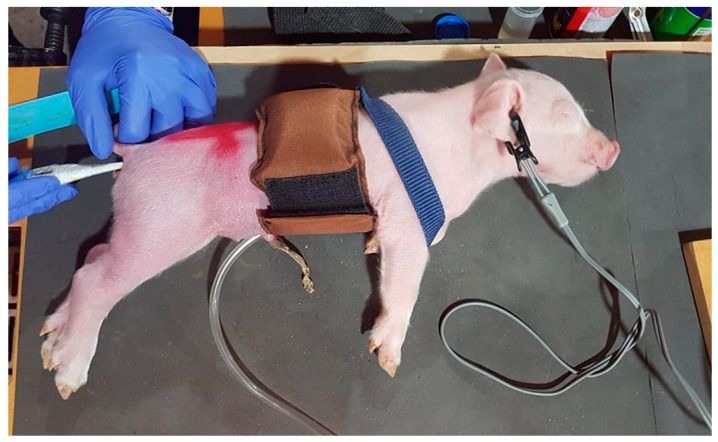
Inflation cuff applied to a piglet, secured around the thorax with velcro and prevented from slipping onto the abdomen by a soft fabric strap. A rectal thermometer is being inserted into the rectum, and a pulse oximetry electrode is attached to the ear.

For application of the inflation cuff, the piglet was held in a standing position while a custom-designed inflatable band was wrapped around its thorax and secured snugly using velcro. The nylon band was 8 cm wide × 36 cm long and housed a rectangular inflatable rubber bladder that extended the length of the cuff to the outer velcro segment. The cuff was held in place around the thorax using a nylon strap with an adjustable clip, placed around the shoulders and neck to prevent slipping onto the abdomen (Figure 2). 

Once the cuff was in place, the bladder was inflated to a maximum pressure of 180 mm Hg using a one-way valve pump with a pressure gauge attached. The timer was started when inflation of the cuff commenced. Release of the squeeze was achieved by deflating the cuff and undoing the velcro and shoulder strap.

### 2.4. Criteria for Discontinuation of the Squeeze

To ensure the safety of the piglets during squeeze application, several physiological variables were qualitatively evaluated at 2 and 8 min after the start of squeeze application (Table 1). Marked changes in any of these parameters were considered to indicate physiological instability and triggered an immediate discontinuation of the squeeze. Piglets were also monitored for any prolonged struggling once the squeeze was applied. If the struggling lasted 2 min, the treatment was discontinued. Likewise, if the piglet was calm during the application of the squeeze, but failed to lose posture, close its eyes and stop moving by 2.5 min (i.e., it failed to be induced), the squeeze was immediately discontinued. The squeeze was also discontinued if the induced animal showed high intensity arousal (Table 2) before the end of the 10 min period. 

### 2.5. Data Collection

Behavioural responses were assessed at two time points (2 and 8 min) following the application of the squeeze according to Table 2, both in real time and by reference to video recordings. Neural reflexes were tested for piglets that were successfully induced into a less-responsive state. Reflexes could not be evaluated on piglets that exhibited high intensity arousals after successful induction or on one piglet that started forceful and increased inspiration indicative of physiological instability, triggering a discontinuation of the squeeze before they could be tested at 2 min. Video and audio were captured using a Sony Handycam (DCR-SR85, Sony Corporation, Tokyo, Japan) placed on a tripod with a full view of the animal.

### 2.6. Data Analysis

The success of the squeeze in inducing a less-responsive state and subsequent maintenance of this state were categorised as follows:

A: No induction of a less-responsive state.

B: Induction of a less-responsive state occurred within 180 s of the start of application of the squeeze but was not maintained for the full 10 min observation period due to high intensity arousals. 

C: Induction of a less-responsive state occurred within 180 s of the start of application of the squeeze and was maintained for the 10 min squeeze duration. 

D: Induction of a less-responsive state occurred within 180 s of the start of application of the squeeze, but the squeeze was discontinued due to concerns based on physiological monitoring. 

The numbers and percentages (with 95% confidence intervals) of animals in each category are presented. The effects of the induction category or the method of squeeze application on latency to induction (loss of posture, closed eyes and cessation of movement) were explored using Kaplan–Meier graphs and log-rank tests. The effects of the induction category or the method of squeeze application on the presence/absence of pedal and palpebral reflexes and the frequency of low intensity arousals during the squeeze were explored graphically and using Fisher’s exact tests and Kolmogorov–Smirnov tests. All summary statistics and graphical representations were produced using R Studio version 1.2.1335 [18]. 

## 3. Results

### 3.1. Success of Induction

All 17 piglets were successfully induced, thus there were no Category A piglets (Table 3). Overall, 8 of 17 (47.1%) piglets did not complete the 10-min squeeze period, seven because of subsequent high intensity arousals (Category B) and one because of physiological instability (Category D). In the rope group, five out of eight squeezes were discontinued, all due to high intensity arousals. Of the nine piglets squeezed with the cuff, the squeeze was discontinued for three piglets, one due to gasping at 265 s and two due to high intensity arousals at 115 and 160 s. The discontinuations always occurred early in the squeeze; piglets that remained less responsive up to approximately 240 s stayed that way for the full 10 min duration (Category C).

### 3.2. Relationship between Induction Category and Time to Induction and Behaviour during the Squeeze

**Figure 3:**
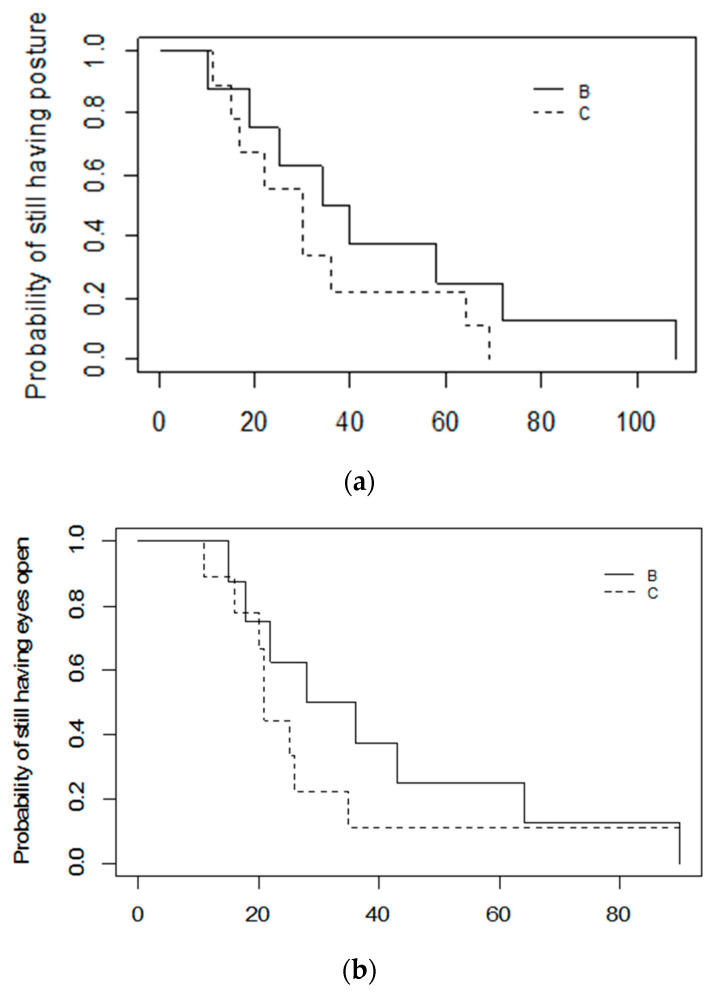

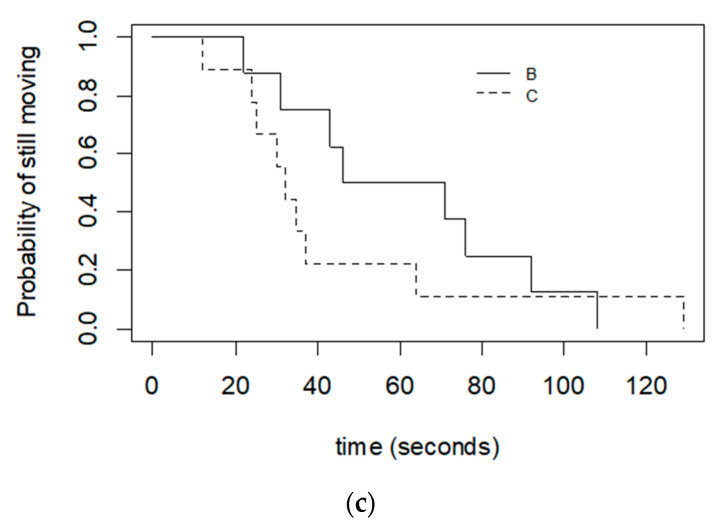
Kaplan–Meier graphs of (**a**) Latency to lose posture, (**b**) Latency to close eyes, (**c**) Latency to cease moving in 17 piglets that were successfully induced into a less-responsive state with either the cuff or the rope, and that did not have the squeeze discontinued for health reasons. Category C piglets (*n* = 9) maintained a less-responsive state for the full 10 min squeeze period, whereas the squeeze was discontinued in Category B piglets (*n* = 7).

The piglets for which the squeeze was discontinued due to high intensity arousals initially lost posture as quickly as the piglets that stayed less responsive for the full 10 min (Cat. B: Median = 37 s, Range = 10–108; Cat. C: Med = 30 s, Range = 11–69; log-rank *p* = 0.30); however, the time to loss of posture was less variable in the Category C piglets (Figure 3a). In contrast, the Category B piglets appeared to take longer to close their eyes and to cease moving than the Category C piglets did. However, statistical tests suggest the difference was not significant for this study population (Latency to close eyes: Cat. B: Med = 32 s, Range = 15–90; Cat. C: Med = 21 s, Range = 11–90; log-rank *p* = 0.30; Latency to cease moving: Cat. B: Med = 58.5 s, Range = 22–108; Cat. C: Med = 32 s, Range = 12–129; log-rank *p* = 0.40) (Figure 3a,b).

**Figure 4:**
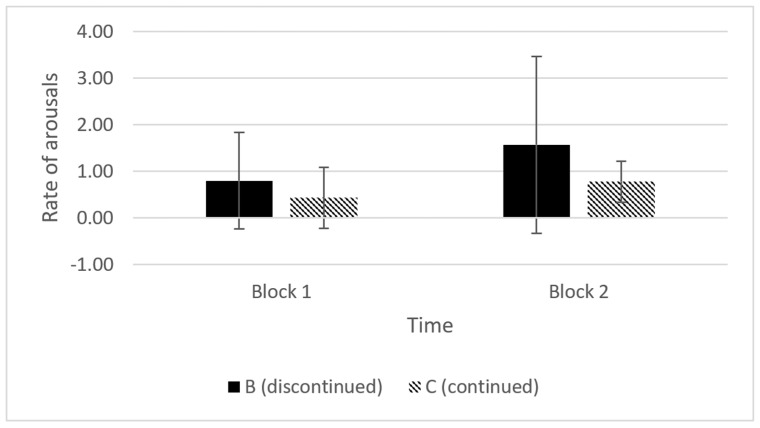
Average (±SD) rate of arousals per 2 min in piglets for which the squeeze was maintained for the full 10 min (Category C, *n* = 9) or discontinued due to high intensity arousals (Category B, *n* = 7), calculated for two time-blocks over the first 4 min of the squeeze. The duration of the first time-block was less than 2 min in some individual piglets, while block 2 was two-minutes long in all piglets. Category B piglets were all discontinued by block 3 (4–6 min) and no comparisons could be made between the two categories after block 2.

The rate of low intensity arousals for the Category B and C piglets could only be compared for the time blocks during which piglets in both groups were still being squeezed, i.e., up to 4 min after application of the squeeze. The rate, rather than actual number, of arousals was compared because the period during which the piglets could arouse in the first 2 min block varied depending on how long induction took. The second block was two min long in all the piglets. The piglets that were subsequently discontinued (Category B) appeared to show a higher rate of low intensity arousals, particularly in block two, than the piglets that remained in a less-responsive state for the full 10 min did (Category C) (Figure 4, Kolmogorov–Smirnov *p* = 0.60). The piglets that subsequently had the squeeze discontinued also showed greater variability in their rate of arousals in block two. However, the statistical test suggests that this difference was not significant in the sample population.

**Figure 5:**
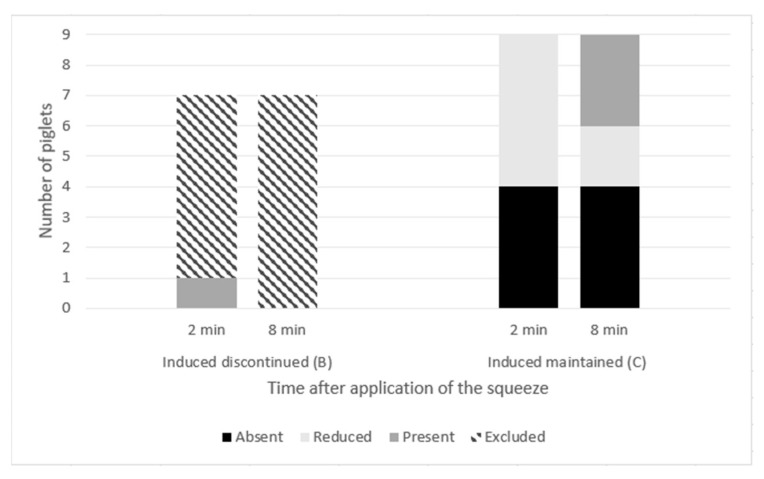
Pedal reflexes of Category B (induced discontinued, *n* = 7) and Category C (induced maintained, *n* = 9) piglets at 2 and 8 min after application of a thoracic squeeze. One piglet that was discontinued for physiological instability (Category D) is not included in the graph. Palpebral reflexes were similar with minor differences in responses for two piglets. Based on their similarities, only pedal reflexes are shown in the graph.

Neural reflexes could not be tested for all the piglets successfully induced. At the 2-min testing point, the squeeze had already been discontinued due to high intensity arousals for three of the seven Category B (Induced discontinued) piglets. Of the remaining four Category B piglets, three could not be tested due to low intensity arousals preceding discontinuation. The one piglet for which reflex testing could be completed showed present pedal and palpebral reflexes at this point. At the 8-min testing point, all seven Category B piglets had already had the squeeze discontinued and could not have reflexes tested. In the Category C (Induced maintained) group, all the piglets had absent (*n* = 4) or reduced (*n* = 5) reflexes at the 2-min testing point. At 8 min, six of the nine category C piglets still had absent (*n* = 4) or reduced (*n* = 2) reflexes and three of the nine piglets had present reflexes (Figure 5, Fisher’s test *p* = 0.16).

### 3.3. Effect of Method of Application on Induction, Discontinuation and Behaviour during Squeeze

**Figure 6:**
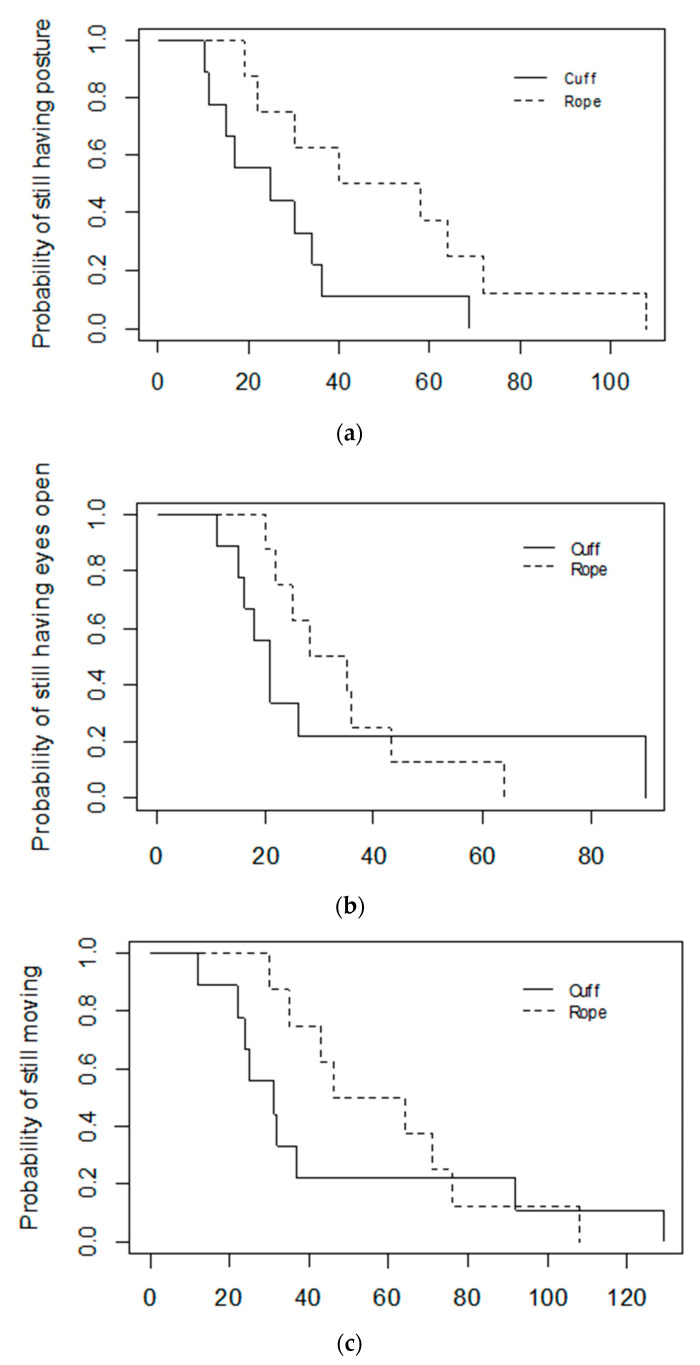
Kaplan–Meier graphs of (**a**) Latency to lose posture, (**b**) Latency to close eyes, (**c**) Latency to cease moving in 17 piglets that were successfully induced into a less-responsive state with either the cuff (*n* = 9) or the rope (*n* = 8).

The latency to lose posture tended to be shorter when the squeeze was applied with the cuff than with the rope (Cuff: Med = 25 s, Range = 10–69; Rope: Med = 49 s, Range = 19–108; log-rank *p* = 0.06), and the time to loss of posture was less variable with the cuff (Figure 6a). Seven out of the nine piglets squeezed with the cuff appeared to close their eyes sooner than the piglets squeezed with the rope (Cuff: Med = 21 s, Range = 11–90; Rope: Med = 31.5 s, Range = 20–64 s; log-rank *p* = 0.70), with the two remaining cuff piglets taking 90 s to close their eyes (Figure 6b). The piglets squeezed with the cuff also appeared to stop moving sooner than the piglets squeezed with the rope (Cuff: Med = 31 s, Range = 12–129; Rope: Med= 55 s, Range= 30–108 s; log-rank *p* = 0.50) (Figure 6c). The piglets for which the squeeze was discontinued, for reasons of physiological instability or because they aroused, apparently did so earlier if they were squeezed with the cuff than with the rope (Cuff: Med = 160 s, Range 115–265, *n* = 3; Rope: Med = 210 s, Range = 165–239, *n* = 5; log-rank *p* = 0.90). However, the statistical tests conducted show no significant effect of the squeeze application method on induction and discontinuation behaviours.

**Figure 7:**
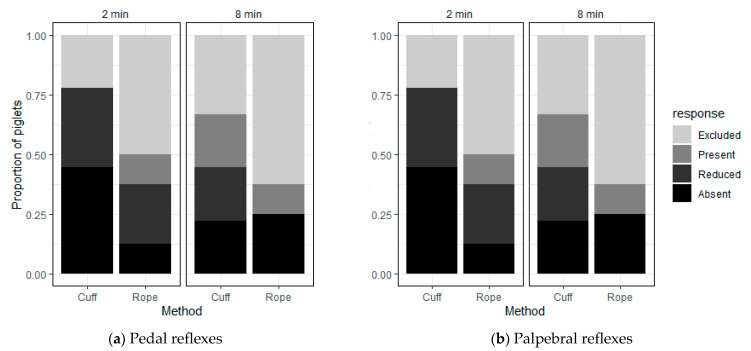
Number of piglets exhibiting each level of (**a**) Pedal reflex responses and (**b**) Palpebral reflex responses at 2 and 8 min after application of the thoracic squeeze for each method of application. ‘Excluded’ refers to piglets that did not have their reflexes tested because of low intensity arousals that preceded a discontinuation of the squeeze soon after the testing point or because the squeeze had already been discontinued. ‘Present’ refers to a full limb withdrawal or blink of the eye; ‘Reduced’ refers to a slight limb withdrawal or twitch of the eye; ‘Absent’ refers to no limb or eye responses to reflex testing.

At the 2-min reflex testing, the squeeze had already been discontinued for two piglets in the Cuff group. Of the seven piglets tested in the Cuff group, all showed either reduced (*n* = 3) or absent (*n* = 4) pedal and palpebral responses (Figure 7a,b; Fisher’s *p* = 0.42 for both pedal and palpebral reflexes). In the Rope group, the squeeze had already been discontinued for one piglet and three of eight piglets could not be tested at that time because of low intensity arousals that preceded a discontinuation of the squeeze soon after the testing point. Two of the four that were tested had reduced pedal responses and one had no response. Palpebral responses were reduced or absent in three out of four.

At the 8-min testing point, the squeeze had already been discontinued in all the ‘excluded’ piglets in both groups. Of those six piglets remaining in the Cuff group at 8 min, pedal and palpebral responses were present in two and reduced or absent in four (Fisher’s test *p*-value = 0.58 for both pedal and palpebral reflexes). In the Rope group, one of the three tested responded, while the other two piglets showed no response for pedal and palpebral reflexes. The statistical tests conducted for pedal and palpebral reflexes show no significant effect of squeeze application method on these variables.

**Figure 8:**
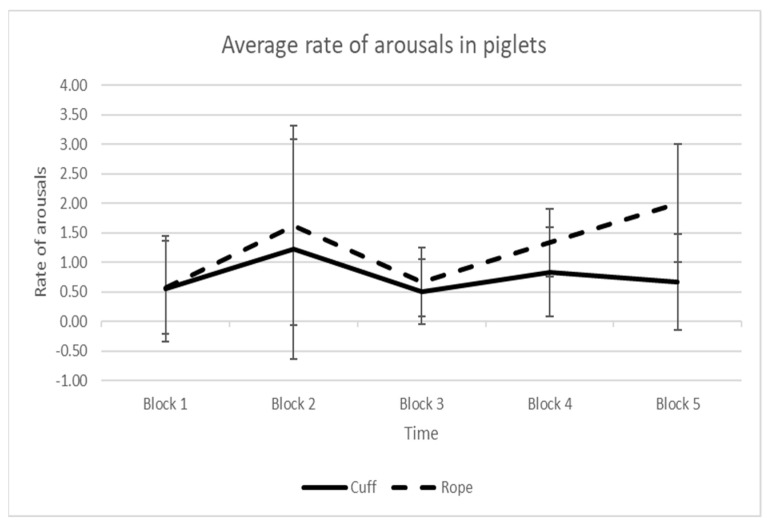
Average (±SD) rate of low intensity arousals during successive 2 min intervals during the full observation period for piglets squeezed with the cuff or rope. The duration of Block 1 varied among individual piglets depending on how long it took for the piglet to be induced, while all other blocks were 2 min long. The number of piglets in each time block decreased from 17 piglets in Block 1 to 9 piglets in Blocks 3, 4 and 5 (*n* = 3 Rope, *n* = 6 Cuff) as the squeeze was discontinued for some piglets.

There appeared to be little difference in the rate of low intensity arousals between application methods, although there was an upward trend in the rate of arousals in the Rope group (*n* = 3) towards the end of the 10-min observation period (Kolmogorov–Smirnov test *p*-value = 0.50) (Figure 8). 

## 4. Discussion

The main aim of this study was to describe the responses of healthy newborn piglets to a standardised application of the thoracic squeeze. A secondary aim was to evaluate the effect of the method of application of the squeeze on those responses. While the outcomes were compared between induction categories and between methods of application, no statistical test produced a *p*-value below the commonly accepted threshold of 0.05. However, this is not surprising considering the low sample size in this exploratory study, and the clinical relevance of differences such as those observed here cannot be dismissed based on hypothesis testing only [19]. Hence, results were interpreted also considering confidence intervals, trends and clinical significance. 

### 4.1. Behavioural Responses

All the piglets were successfully induced into a ‘less-responsive’ state, characterised by a loss of posture, closed eyes and cessation of movement. These results are consistent with previous reports of the responses of healthy foals and low vigour foals and calves to a thoracic squeeze [9,10]. All the foals and calves squeezed were reportedly successfully induced into a less-responsive state characterised by lateral recumbency and cessation of movement [9,10]. The observed responses of healthy piglets to thoracic squeezing suggests that this phenomenon can be generalised to neonates of other precocial mammalian species.

While all the piglets were successfully induced, seven (41%) aroused during the squeeze, which was subsequently discontinued. This arousal always happened early in the squeeze (<4 min), suggesting that these piglets were not fully induced into a less-responsive state despite showing induction behaviours. In support of this, pedal and palpebral responses were present in one piglet that was still less responsive at 2 min, but that subsequently aroused. In contrast, the piglets that maintained the less-responsive state for the full 10 min all had reduced or absent reflexes at 2 min. At 8 min, two-thirds of these Category C piglets still showed significant behavioural and reflex suppression, whereas all the Category B piglets had become fully responsive. Furthermore, consistent with these observations, the piglets that aroused during the squeeze generally took longer to be induced than those that maintained the less-responsive state for the full 10 min, with a longer latency to close their eyes and stop moving once recumbent. They also showed more low intensity arousals before the discontinuation of the squeeze than the Category C piglets did.

These data suggest that there were two cohorts of piglets that responded differently to the thoracic squeeze. Some piglets (Category B), while successfully induced, may have not been fully induced into a less-responsive state, with persistent pedal and palpebral reflexes indicating they were still responsive to external stimuli [20]. In contrast, about half of the induced piglets may be considered truly induced into a behavioural and neurally less-responsive state, with reduced or absent reflexes. These observed changes in reflexes are similar to those reported for animals under a moderate plane of chemical anaesthesia, supporting the notion that these piglets were truly induced into a less-responsive state during the squeeze [20,21]. However, even in those piglets that maintained a less-responsive state, there were signs of ‘recovery’ during the 10-min period of the squeeze. One third of the Category C piglets (*n* = 3) showed evidence of a return to normal reflex responses at 8 min after the application of the squeeze. This suggests that they were returning to a more responsive state as the time after the application of the squeeze progressed. 

### 4.2. Potential Mechanisms Underlying the Thoracic Squeeze

The mechanisms underlying these behavioural responses to the thoracic squeeze are unknown. However, there is no evidence that this is a fainting event. Fainting refers to a sudden and brief loss of posture and consciousness caused by a significant decrease in cerebral blood pressure [22]. Typical signs of fainting involve a sudden change in cardiovascular function (increased breathing rate, decreased blood pressure and blood oxygen levels, increased heart rate) that suggest reduced blood flow to the brain [23,24]. In this study, the physiological variables (heart rate, respiratory rate, mucous membrane colour, muscle tone and, where possible, oxygen saturation levels) monitored throughout the squeeze indicated that the piglets were physiologically stable. The one piglet for which the squeeze was discontinued due to gasping did not show marked changes in cardiovascular function such as heart rate, oxygen saturation or colour of the oral mucosa. Moreover, the loss of posture consistent with fainting had already occurred during induction. Upon the application of the squeeze, all the piglets switched from thoracic to abdominal breathing, but breathing depth and rate and heart rate did not change substantially and there was no indication of a decrease in blood oxygen levels. The general physiological stability of the piglets in this study, therefore, suggests that fainting was unlikely to be the cause of the less-responsive state observed.

The response to the squeeze resembles those characterised as Tonic Immobility. Tonic Immobility (TI) refers to a temporary state of motor inhibition and behavioural quiescence, often elicited by certain forms of restraint and reported in vertebrates of numerous species and ages [25]. Reported responses vary considerably, but the general characteristics of TI are reduced responsiveness following induction by restraint and motor quiescence [26]. 

The induction of TI is partly attributed to a neural reflex, based on the speed at which the animals become immobile [27,28,29]. In the current study, induction always occurred within 2.5 min of the start of the squeeze application and usually much more quickly. Importantly, this is the first study to characterise the speed of induction after the application of a thoracic squeeze. Based on the observed speed of induction, it is possible that a neuroinhibitory reflex is responsible for the response of neonatal mammals too. 

The mechanisms of TI are understood to involve forebrain and brainstem structures that, when activated, profoundly inhibit motor output, cause autonomic changes such as increased or decreased heart rate, respiratory rate and blood pressure and decreased responsiveness to external stimuli [25,30]. Importantly, marked increases in hypothalamic-pituitary-adrenal (HPA) activation appear to be associated with induction into TI and maintenance of the state, leading to the interpretation of TI as a fear or stress response [31,32]. In support of this interpretation, previous research applying a squeeze to healthy foals found increases in cortisol and adrenocorticotropic hormone (ACTH) levels associated with changes in cardiovascular function during the squeeze [9]. While the responses of piglets to a thoracic squeeze appear to be similar to TI, further research is required to determine whether the mechanisms are the same. In particular, there is a need to explore the changes in the HPA activation of piglets during the squeeze, as well as forebrain and brainstem activity during induction. This is key to determining whether the squeeze would be appropriate for therapy or lower-stress handling and restraint of neonatal farm mammals.

One additional theory has been proposed regarding the mechanism underlying the observed responses of neonatal mammals to such squeezing of the thorax. Based on the proximity of the response to the birth, it has been proposed that there may be specific birth-related neuroinhibitory factors involved, such as those believed to keep the foetus unconscious and behaviourally quiescent before birth [6,33]. Further research is required to determine whether the response to thoracic squeezing becomes extinct in older animals, which would provide support for a specific birth-related mechanism.

### 4.3. Comparison of the Cuff and Rope Methods

Both the inflation cuff and the rope induced a less-responsive state in all piglets within approximately 2 min. The cuff seemed to induce the piglets faster, with a quicker loss of posture, eye closure and cessation of movement. These results may largely be explained by the faster and more uniform application of the cuff than application with a rope. Due to resistance in the rope, it was difficult to apply the squeeze evenly. As a result, the squeeze could not be standardised for all the piglets and each loop had to be individually tightened, increasing the application time. 

In addition, more piglets aroused when squeezed with the rope (71%). However, the arousal occurred later than in those squeezed with the cuff, which is likely a consequence of the time to induction being longer due to difficulties with application of the rope. More piglets squeezed with the cuff showed reduced or absent reflexes compared to the piglets squeezed with the rope. These observations suggest that the inflation cuff not only induces a less-responsive state faster in piglets but is also better at maintaining that state in piglets. Furthermore, the amount of pressure applied using the cuff was controlled and recorded (mmHg), resulting in less variation between operators and between individual piglets. In contrast, the pressure applied using the rope method could not be recorded and would likely have variation between operators and individual animals based on the type of rope used, tightness, friction and varying pressure applied to the rope by operators. It is, therefore, recommended that the cuff be used to apply the squeeze in future research.

One piglet in the Cuff group had the squeeze discontinued due to evidence of physiological instability after approximately 4.5 min. There were no marked changes in heart rate or breathing rate, but the piglet started gasping, characterised as open mouthed inspiration with increased duration and force [34,35]. Upon the immediate discontinuation of the squeeze, the piglet was observed to recover quickly. This suggests that while the squeeze is generally safe to apply to healthy piglets, close monitoring of the neonates should be recommended. 

### 4.4. Possible Applications for the Thoracic Squeeze

The findings of this preliminary study suggest that there are applications for the thoracic squeeze in an industry setting. The less-responsive state induced in the piglets and maintained for at least several minutes may be useful as a therapeutic approach for neonatal mammals of precocial species that show low vigour behaviours as has been reported in foals and calves [6,10]. Another possible use for the thoracic squeeze technique is as a non-chemical form of restraint, which could be useful for the purpose of conducting quick husbandry procedures such as tail docking and teeth clipping in neonates. However, more information is required to determine whether induction itself causes stress and whether piglets in a less-responsive state experience the same degree of pain as those in a normal responsive state before this method can be applied in an industry setting.

## 5. Conclusions

The thoracic squeeze elicited a less-responsive state in all piglets in this study, characterised by loss of posture with eyes closed and no movements of the head, neck or limbs. More than half of the induced piglets stayed in the less-responsive state for the full 10-min squeeze period. Those that aroused did so early, showed differences in the timing of behaviours at induction and exhibited more low intensity arousals in the lead-up to full arousal and the discontinuation of the squeeze. Thus, it may be possible to identify individuals likely to arouse, which is useful to know for practical applications of the squeeze such as restraint for husbandry procedures.

The inflation cuff appeared more practical in its application and, therefore, at inducing and maintaining piglets in a less-responsive state than the rope and was faster and easier to apply consistently and to remove. Thus, it is recommended that the inflation cuff be used in future research. 

These findings suggest that the less-responsive state previously reported in foals undergoing a thoracic squeeze in the early post-natal period may represent a generalised phenomenon in neonates of precocial mammalian species. The thoracic squeeze has been demonstrated to be generally safe for inducing and maintaining a less-responsive state in apparently healthy piglets, although close physiological monitoring is recommended before, during and after the squeeze and the response may be brief in some individuals. This preliminary study provides a foundation for further research using the inflation cuff to explore the mechanisms underlying the thoracic squeeze and ways in which the squeeze can be effectively applied in an industry context. 

## Figures and Tables

**Table 1:** Physiological variables evaluated during (2 and 8 min after application) and after the 10 min squeeze to check initial health, stability during the squeeze and recovery afterwards.

Variable	Description
**Heart rate**	Determined as beats per minute using a stethoscope placed on the chest between the forelimbs for 30 s.
**Rectal temperature**	Determined as °C using a lubricated thermometer (Shoof digital thermometer T series) inserted into the rectum.
**Oxygen saturation**	Determined, when possible (%), using a pulse oximeter (Coviden Nellcor PM10N) attached to an ear.
**Respiration rate**	Determined as breaths per minute by visual assessment of chest movement for 30 s.
**Muscle tone**	Assessed qualitatively as rigid or relaxed, by moving and bending the forelimb and hindlimb joints. Muscles were evaluated as rigid when there was a large amount of resistance and relaxed when there was no resistance to limb movement.
**Posture**	Assessment of body posture, determined as standing or lying in lateral recumbency.
**Head position**	Position of the head while in lateral recumbency, determined as raised or flat on the lying surface.
**Eyes**	Eye status determined as open or closed.
**Oral mucosa colour**	Determined as pink or blue by visual assessment of the gums and tongue.

**Table 2:** Behavioural responses recorded during application and maintenance of the 10 min thoracic squeeze.

Variable	Description
**Latency to loss of posture**	Time to enter lateral recumbency from a standing position.
**Latency to eye closure**	Time to close eyes, with eyes remaining closed for at least 5 s.
**Latency to cessation of movement**	Time to the cessation of all limb and head movements.
**Pedal reflex**	Status of reflex determined by pinching the interdigital cleft between the claws of a front foot to elicit withdrawal of the limb. Present was assigned for a full limb withdrawal response; reduced for a slight limb withdrawal or a limb twitch response; and absent for no muscle twitch or limb movement.
**Palpebral reflex**	Status of reflex determined by lightly brushing or touching the eyelashes or skin in the lateral corner of the eye, to elicit a blink or eye twitch response. Present was assigned for a full twitch or blink response; reduced for a small twitch response; and absent for no muscle twitch.
**Low intensity arousal**	Low intensity, short duration movements. Characterised by eye opening and vigorous limb and head movements for 10 s or less, before a less-responsive state was resumed.
**High intensity arousals**	High intensity movements, lasting longer than 5 s. Characterised by opening of the eyes and vigorous movements with righting onto all four feet.

**Table 3:** Number and percentage of piglets in each induction category for each method of squeeze application, with 95% confidence intervals for the percentage in each category. Category A refers to piglets that were not induced into a less-responsive state; Category B refers to piglets that were induced but had the squeeze discontinued due to high intensity arousals; Category C refers to piglets that were induced and maintained in a less-responsive state for the full 10 min; Category D refers to piglets that were induced but discontinued due to physiological instability.

Category	A (Not Induced)	B (Induced but Discontinued)	C (Induced and Maintained)	D (Induced but Unstable)	Total
Rope	0	5	3	0	8
Cuff	0	2	6	1	9
Total	0	7	9	1	17
Percentage (95% CI)	0% (0–20%)	41.2% (18–67%)	52.9% (28–77%)	5.9% (15–29%)	100%

## Data Availability

Contact the corresponding author.
